# Think globally, act locally: Phylodynamic reconstruction of infectious bronchitis virus (IBV) QX genotype (GI-19 lineage) reveals different population dynamics and spreading patterns when evaluated on different epidemiological scales

**DOI:** 10.1371/journal.pone.0184401

**Published:** 2017-09-07

**Authors:** Giovanni Franzo, Paola Massi, Claudia Maria Tucciarone, Ilaria Barbieri, Giovanni Tosi, Laura Fiorentini, Massimo Ciccozzi, Antonio Lavazza, Mattia Cecchinato, Ana Moreno

**Affiliations:** 1 Department of Animal Medicine, Production and Health (MAPS), University of Padua, Legnaro, Padua, Italy; 2 Sezione di Forlì, Istituto sperimentale della Lombardia e Emilia Romagna, Forlì, Forlì Cesena, Italy; 3 Department of Virology, Istituto sperimentale della Lombardia e Emilia Romagna, Brescia, Italy; 4 Department of infectious, parasitic and immune-mediated diseases, Istituto Superiore di Sanità, Roma, Italy; Sun Yat-Sen University, CHINA

## Abstract

*Infectious bronchitis virus* (IBV) represents one of the poultry industry major threats, particularly in high density producing countries. The emergence and spread of new IBV genotypes have frustrated the various disease control efforts implemented over time. Despite that, few comprehensive and large scale studies have been performed to understand the international and local spreading dynamics of this virus. In the present work, these phenomena were evaluated by implementing a Bayesian phylodynamic approach to reconstruct the epidemiological patterns and population history of the QX genotype (currently renamed GI-19 lineage), the most relevant IBV lineage of the Old-World. Our analysis, based on 807 partial S1 sequences of strains collected from 18 countries between 1993 and 2015, demonstrates that this genotype originated in China well before its first identification. After a prolonged local circulation, it started spreading to other European, Asian and Middle East countries in successive waves, which were mirrored by concomitant fluctuations in viral population size. Interestingly, the within-Europe spread was characterized by a higher estimated migration rate compared with the inter-continental one, potentially reflecting the closer geographic and economic relationships among these countries. Nevertheless, the colonization of new states by the GI-19 lineage appeared to occur mostly by single introduction events in both intra and inter-continental spread, likely because of epidemiological factor and health policy combination which seems to prevent the frequent introduction and mixing of different strains. On the other hand, the within Italy QX circulation reconstruction showed a much more intricate connection network among different locations, evidencing the difficulty in controlling IBV spread especially in highly densely poultry populated areas. The presence of several well supported epidemiological links among distantly related Italian regions testifies that animal transportation and indirect transmission routes rather than local airborne diffusion contribute to the QX success and persistence at local scale. Globally, the spreading dynamics and evolution of the QX genotype were reconstructed from its very origin to nowadays, demonstrating the need of more effective direct control measures, particularly within each country. Unfortunately, the incompleteness of available molecular epidemiology data represents an insurmountable limit which leaves many questions currently unsolved, thus highlighting the compulsoriness of a structured monitoring and data sharing system implementation.

## Introduction

*Infectious bronchitis* (IB) represents one of the most relevant viral diseases of poultry and it is responsible for remarkable economic losses all over the world due to both direct and indirect costs. In fact, IBV causes mainly a highly contagious upper respiratory tract disease, with a morbidity of typically 100% and mortality reaching 50% when some strains that cause nephritis or opportunistic infections occur [1]. Additionally, the genital tract of layer and breeder birds can also be affected, causing reproductive disorders and altered egg production [2].

The infection is caused by a single-stranded positive-sense RNA virus belonging to the order *Nidovirales*, family *Coronaviridae* genus *Coronavirus*. The viral genome is about 27 Kb long and encodes for different proteins, such as the RNA-dependent RNA polymerase (RdRp), numerous accessory and regulatory proteins and the structural proteins spike, envelope, membrane, and nucleocapsid [1,3]. Particularly, the spike protein (S) (or part of it) is widely studied and sequenced because of its importance in the viral life cycle, in the development of the host immune response and as an epidemiological marker. This protein is post-translationally cleaved in the S1 and S2 subunits. The former contains domains for cell receptor attachment while the latter has a transmembrane domain anchoring the spike to the virion. Besides being involved in viral adhesion, membrane fusion and viral entry, the S protein contains epitopes inducing neutralizing antibodies [3] and therefore its variability is essential in conditioning the cross-protection among strains [4]. As other RNA viruses, IBV is characterized by a high substitution [5,6] and recombination rate [7,8], which are responsible for the continuous emergence of new genetic and antigenic variants characterized by heterogeneous biological and epidemiological properties. While in the past IBV characterization depended on virus isolation and serological assays (i.e. virus neutralization), the advent of molecular biology techniques has radically changed the routine typing approach. Despite the not direct genotype-serotype relationship, the currently adopted IBV classification and nomenclature are based on the genotypic features and strains are therefore grouped in so called *“genotypes”*. Unfortunately, there was no agreement on the exact method by which sequences should have been compared and on the criteria for differentiating and naming the genotypes.

Recently, Valastro et al., [9] defined a new and coherent classification scheme based on the S1 sequence phylogenetic analysis, dividing IBV strains in 32 lineages grouped in 6 genotypes. To avoid misconceptions, it must be stressed that in the current manuscript the former concept of *genotype* will be expressed by the *lineage* term, according to the aforementioned classification [9].

Interestingly, while the vast majority of variants remained confined in time and space, some viruses have managed to invade broader regions or even to emerge as a worldwide threat [4,9,10].

Among the latter, the GI-19 (previously referred as QX genotype) probably represents the most relevant IBV lineage of the Old-World. Firstly described in China in 1996 [11,12], it has then spread westward invading Russia [13,14], Middle East [15–17] and Europe [1,18–25], becoming the most prevalent field strain in many countries [26].

Initially associated with proventriculitis outbreaks [12], over time it has been demonstrated to be responsible for respiratory signs, nephritis and reproductive disorders, including the false layer syndrome [4,27,28]. Besides the direct cost due to the reduced productivity and mortality, additional accessory costs had to be sustained by poultry companies to control the disease.

Several experimental [29] and field [21] studies confirmed the efficacy of vaccination schemes based on the protectotype concept [30]. However, the rapid spread and high virulence [27] of this new variant challenged the previously established control strategies and has forced their re-assessment and the development of new homologous vaccines. l [31]. This massive impact has prompted the implementation of several epidemiological studies in different countries in the attempt to better understand the rapidly evolving scenario. Nevertheless, the local nature of many of these studies and the lack of a properly planned sampling made difficult to define the history and the trajectories followed by this virus from its origin to nowadays. Particularly, little information is available about its population dynamics over time and the major routes of its international spread.

To cover this lack of information, a large scale phylodynamic study has been performed based on the S1 protein hypervariable region analysis of strains sampled all over the world, from the first GI-19 lineage (alias QX genotype) detection to the end of 2015. Additionally, to better understand the GI-19 lineage fate after its introduction in a previously naïve county, the spreading and population variation patterns were evaluated with reference to Italy, a major poultry producing country, where, after the first description in 2005, this lineage established itself as the predominant one [21].

## Material and methods

### Italian strain detection and sequencing

Samples of the trachea, kidneys and cloacal saws were collected from suspected IBV-infected chickens and submitted to the diagnostic laboratories of the Istituto zooprofilattico della Lombardia e Emilia Romagna for routine diagnosis. The samples were delivered from the whole country but most of them were collected from the highly densely populated regions of Northern Italy. Viral RNA was extracted from clinical samples using the TRIzol reagent (Invitrogen, Carlsbad, CA, USA) according to the manufacturer’s protocol. IBV was detected using RT-PCR as we previously described [8]. Partial sequencing of the S1 gene (464-bp) was performed using the same primers that were used for RT-PCR [32].

The obtained chromatogram quality was evaluated by FinchTV (http://www.geospiza.com) and the consensus sequences were reconstructed using CromasPro (CromasPro Version 1.5).

### QX database preparation/definition

All complete and partial IBV S1 sequences whose collection country and date were available (1808 sequences) were downloaded from Genbank. These sequences plus the Italian ones were merged with the reference set provided by Valastro et al., [9] and aligned using MAFFT [33]. To obtain a dataset representative of a relevant number of countries and time periods, guaranteeing at the same time the consistency in term of alignment coverage, only the highly sequenced S1 hypervariable region was maintained; the remaining regions were trimmed and the strains for which this portion was not available were removed. Additionally, poorly aligned sequences and those with premature stop codons, suggestive of sequencing errors, were removed from the alignment.

A phylogenetic tree was then reconstructed using PhyML [34] selecting as the most appropriate substitution model the one with the lowest Bayesian Information Criterion (BIC) calculated using Jmodeltest [35]. The phylogenetic tree was used to classify the strains and those potentially belonging to the GI-19 group (QX genotype) were extracted. The presence of recombinant strains was evaluated using RDP4 [36] on a dataset including the selected GI-19 like strains plus the reference ones. The RDP4 settings for each method were adjusted accounting for the dataset features according to the RDP manual recommendations. In particular RDP, GENECONV, Chimaera and 3Seq were used in a primary scan while the full set of available methods was used for the analysis refinement. Only recombination events detected by more than 2 methods with a significance value lower than 10^−5^ (p- value < 10^−5^) and Bonferroni correction were accepted. After recombinant removal, the absence of residual breakpoints was confirmed with SBP [37] implemented in HyPhy [38]. Finally, TempEst [39] was used to assess that heterochronous sequences under investigation displayed sufficient ‘temporal signal’ for reliable analysis. After preliminary checking the molecular clock hypothesis, the sequences whose sampling date was inconsistent with their genetic divergence and phylogenetic position were removed. Indeed, significant deviations from the expectation can be due to several factors including low sequencing quality, excessive passaging leading to the accumulation of cell-line adaptations, recombination, mislabelling of the sequence, sequencing of a vaccine virus (which undergoes no evolution while in storage), etc. [39].

### International dataset

A final international dataset of 807 sequences, including the 454 Italian ones, was considered (S1 Table). To avoid a bias due to unbalanced sequence availability among countries [40], 10 different sequence datasets were created by randomly selecting a maximum of 10 sequences for each country-year pair. All datasets were analysed using the same approach; several viral population parameters (e.g. Time to most common recent ancestor (MRCA), evolutionary rate, population dynamics over time, etc.) were jointly estimated using the Bayesian serial Coalescent implemented in BEAST 1.8.2 [41].

The nucleotide substitution and the clock models were selected respectively using the BIC calculated with jmodeltest [35] and the Bayes Factor (BF) value, calculated estimating the marginal likelihood of the different models using the path sampling (PS) and stepping stones (SS) methods [42]. The population dynamic variations over time were reconstructed using the Bayesian non parametric Skygrid method [43]. This method estimate a composite value (Ne*t), representative of the product between Effective population size (Ne) and generation time (t) [44].

To evaluate the international spreading of GI-19, a discrete state phylogeographic approach was used, assuming each country as an additional discrete feature of the viral strain [45]. By implementing a Bayesian approach it was possible to estimate the ancestor of these traits (i.e. the migration history) jointly with all the other model parameters, thus accounting for phylogenetic and Markov model parameter uncertainty, and to obtain at the same time a reconstruction scaled in natural time, calibrated under the implemented molecular clock and population model. More specifically, an asymmetric substitution model with Bayesian stochastic search variable selection (BSSVS) was selected to model the transition among discrete traits (i.e. countries). Additionally, the implementation of the Bayesian BSSVS allows a BF test that identifies the most parsimonious description of the spreading process [45].

For each dataset a 100 million generation Markov chain Monte Carlo run was performed. Results were analysed using Tracer 1.6 [46] and accepted only if the estimated sample size (ESS) was greater than 200 and the convergence and mixing were adequate. Parameter estimation was summarized in terms of mean and 95% Highest Posterior Density (HPD) after the exclusion of a burn-in equal to 20% of the run length. Maximum clade credibility (MCC) trees were constructed and annotated using Treeannotator (BEAST package).

SpreaD3 [47] was used to display the spreading process over time and to calculate the BF associated to each migration route. The directional transition rates among countries were considered non-zero (i.e. significant) when the BF was greater than 20. Additional summary statistics were calculated for the different runs using home-made specific R scripts [48].

### Italian dataset

Out of the whole Italian QX database, only the sequences belonging to the monophyletic group including (with few exceptions) exclusively Italian strains were considered in the current analysis. A dataset of 454 sequences was obtained (S2 Table). This approach was necessary to focus the attention on strains that continuously circulated and evolved in Italy after QX introduction (i.e. excluding recently imported strains). Otherwise, the population parameter estimation (e.g. MRCA, population dynamics, etc.) would have mirrored those of a broader geographical area and viral population, falsifying the results.

An approach fully comparable with the previously described one (see previous paragraph) was used to reconstruct the GI-19 history in Italy. The minor differences are herein summarized:

1. regions instead of countries were selected as discrete states for the phylogeographic analysis.
2. when the ten sub-datasets were created, 20 sequences (instead of 10) were selected for each region-year pair.
3. to improve the resolution of the migration pattern reconstruction, the discrete state phylogeography was performed on the whole Italian sequence dataset (the monophyletic group). A constant size population model was selected to reduce the computational burden and improve the mixing. The plausibility of this assumption was evaluated by inspecting the results of the non-parametric Skigrid estimated during the previous runs (see point 2)).

## Results

### Datasets

After sequence filtering a final international dataset was obtained, including 807 partial S1 sequences of strains collected from 18 countries between 1993 and 2015 (S1 Table). TempEst analysis demonstrated the presence of a significant temporal structure, being the correlation between genetic distances and sampling dates equal to 0,57 (R^2^ = 0,33; slope 2,96·10^−3^). Additionally, the Xia test, performed for each codon position, demonstrated the absence of substitution saturation. The obtained dataset was thus considered suited for further analysis.

The Italian dataset, after filtering and removal of recently imported strain, included 454 sequences for which collection region and date were available. Strains were sampled from 16 regions between 2005 and 2015 (S2 Table).

#### Intentional dataset

**Fig 1:**
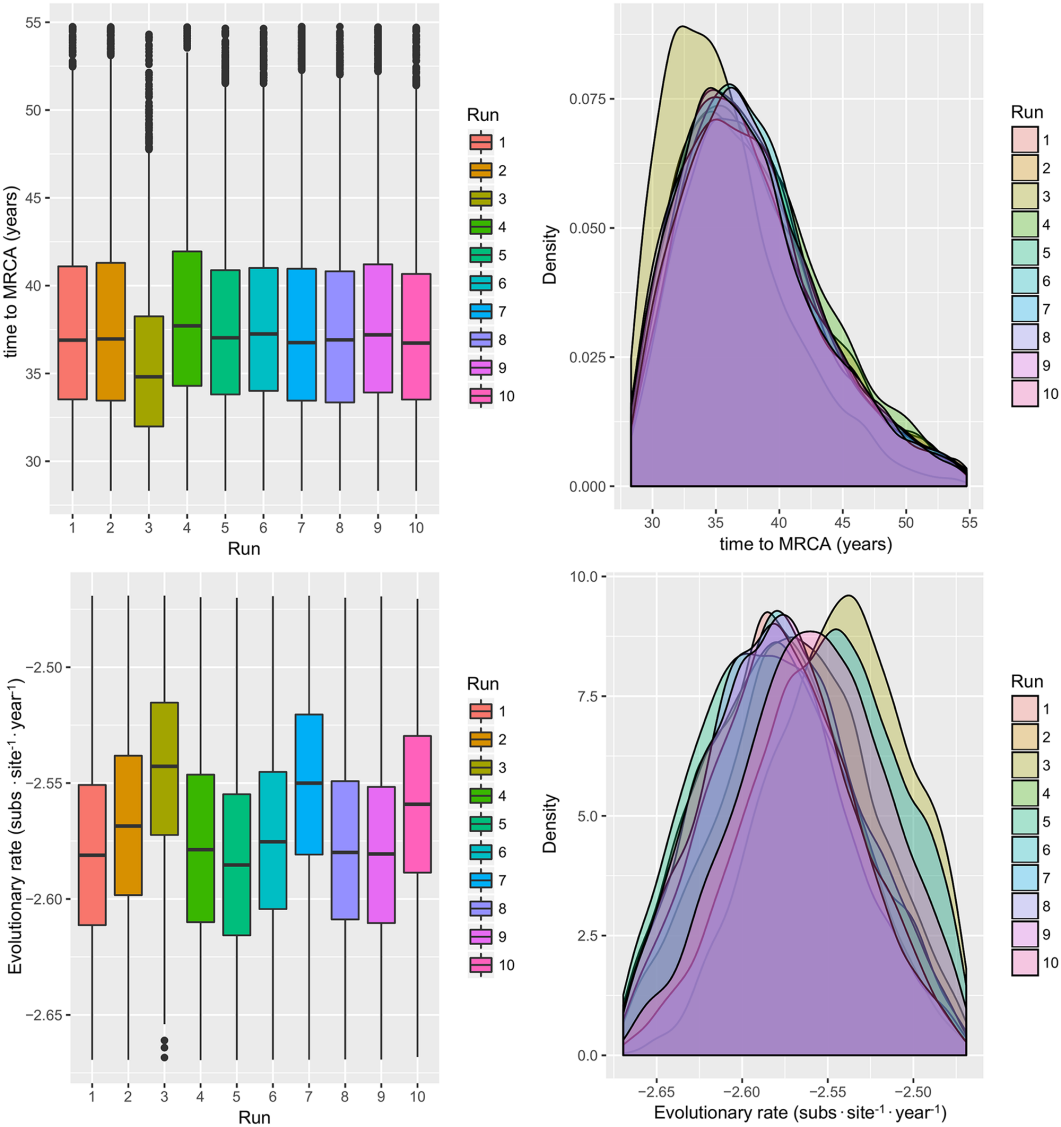
QX genotype MRCA and evolutionary rate. Upper figure: Boxplot (left) and Densityplot of the MRCA posterior probability. Lower figure: Boxplot (left) and Densityplot of the mean evolutionary rate (expressed in base-10 logarithm) posterior probability. Results have been estimated performing ten independent runs based on sequences randomly sampled from the international database. The 95HPD intervals are reported for both figures.

**Fig 2:**
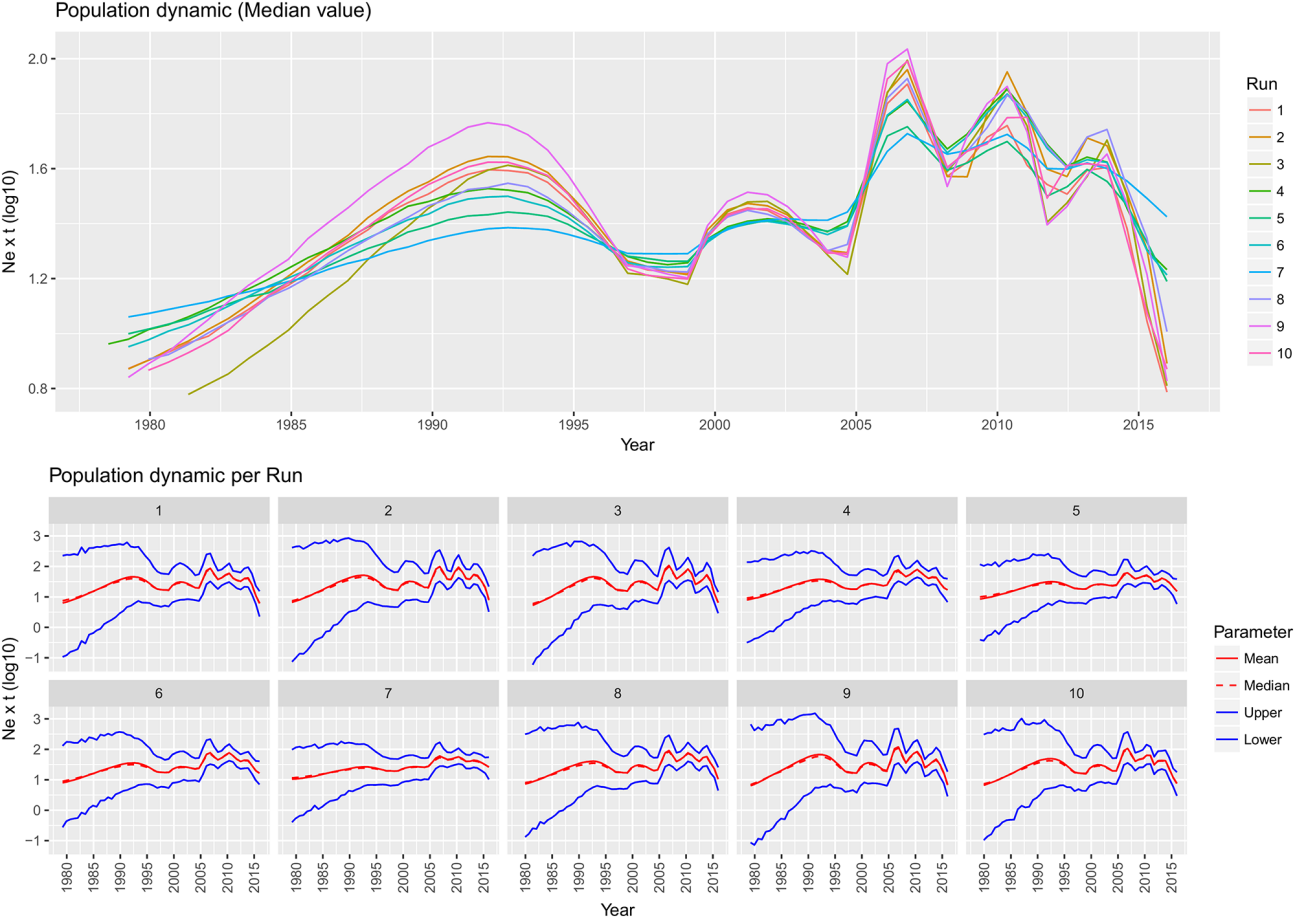
QX genotype population dynamics. Upper figure: Mean relative genetic diversity (Ne x t) of the worldwide GI-19 population over time. The results of the ten independent runs have been color-coded. Lower Figure: Mean, median and upper and lower 95HPD values are reported for each run.

BEAST analysis performed on the 10 randomly generated datasets showed concordant results in terms of MRCA, substitution rate (Fig 1) and viral population history (Fig 2). Mean MRCA and substitution rate over the 10 run were 1977,86 [95HPD:1965,54–1986,64] and 2,71·10^−3^ [95HPD:2,22·10^−3^–3,26·10^−3^], respectively.

The population dynamics analysis revealed that after the MRCA the viral population increased until about 1993. Thereafter, the relative genetic diversity fluctuated over time, with at least four distinct peaks that followed one another until the end of the considered time period, when a more significant decrease was observed.

**Fig 3:**
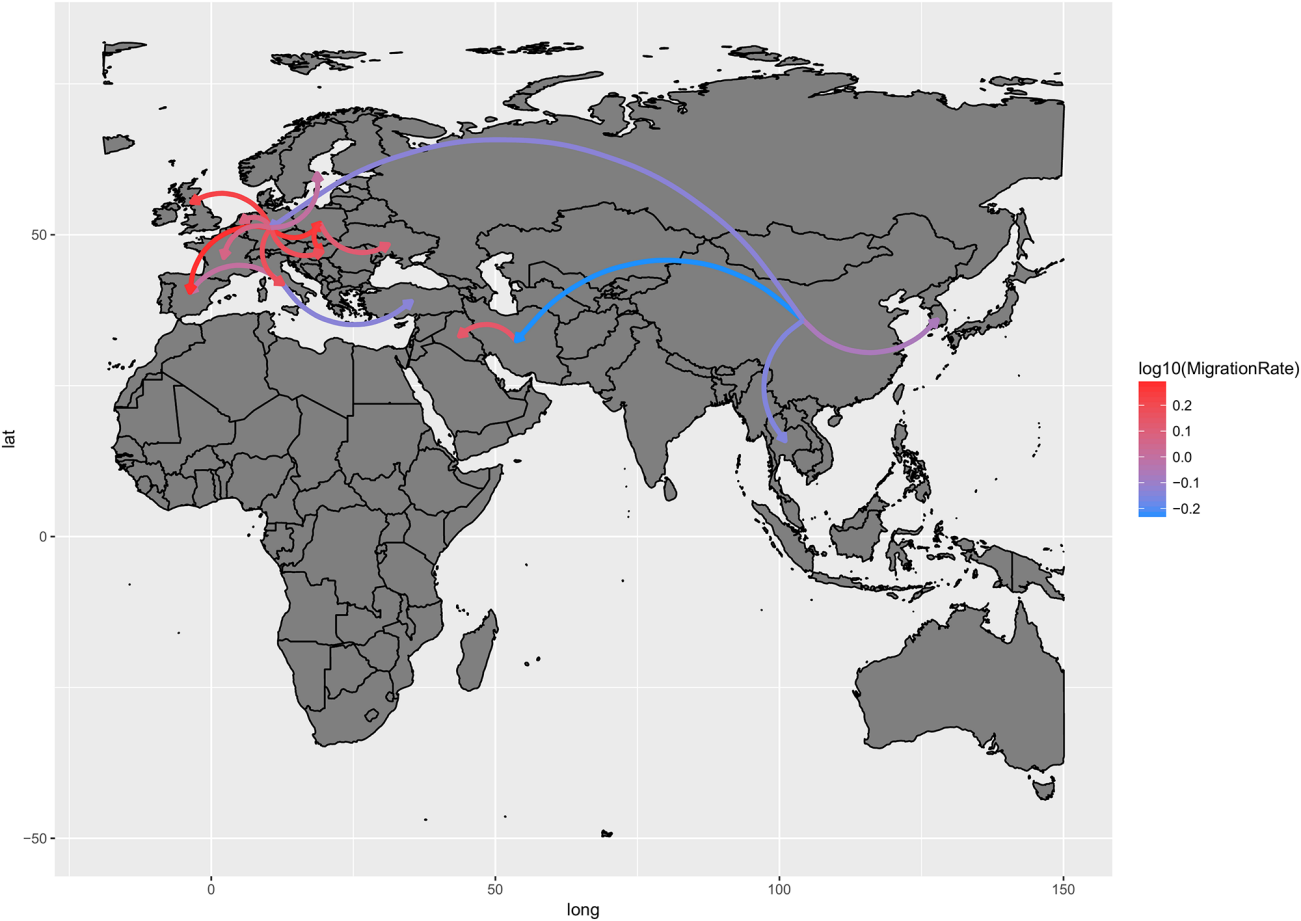
QX genotype migration paths. Well supported migration paths (i.e. BF>20) among countries are depicted. The arrows indicate the directionality of the process while the edge colour is proportional to the base-10 logarithm of the migration rate. The location of each country has been matched with its centroid.

The migration rate analysis revealed different well supported spreading path (Fig 3). Remarkably, the same routes were identified as significant using the ten randomly built datasets with the only exception of Germany to Sweden and South Africa to China connections that were identified only in run 4 (S1 Fig). Considering the robustness and the consistency of the obtained estimates, only the results of run 2, which showed the higher ESS and the best mixing, will be described for graphical and interpretability reasons. It is possible to recognize two nuclei of global spread: China and Europe. The former represented the original epidemic source and contributed to the spread to Europe, other Asian countries and Middle East. After introduction in Europe, most likely in Germany, the virus widely circulated in this area, as evidenced by the higher migration rate among the UE countries compared to other ones (Fig 3). Countries of the central Europe played a relevant role in the dissemination to Eastern Europe ones while Mediterranean countries seemed to contribute to the GI-19 lineage spread to Northern Africa (Egypt) and Turkey.

A more detailed picture of the GI-19 history was obtained by plotting the MCC tree in time and space. After its origin in China in the late '70, the GI-19 lineage circulated within the country until the late '90 when it migrated to Europe (Germany; ~2002) and Thailand (~2005). After European first introduction, it rapidly spread to all main countries during the first half of the decade: Poland and Italy (~2003) and the Netherlands and France (~2004). Then, Spain (~2006), Sweden and United Kingdom (~2007), Ukraine (~2009) and Hungary (~2010) were involved. In the following years, Europe contributed to the disease spreading to other countries; France to South Africa (~2010), Italy to Turkey (~2011) and Spain to Egypt (~2015). In the meanwhile, strains from China invaded South Korea (~2008) and Iran (~2011) from where they spread to Iraq (~2013) (S1 Video).

#### Italian dataset

**Fig 4:**
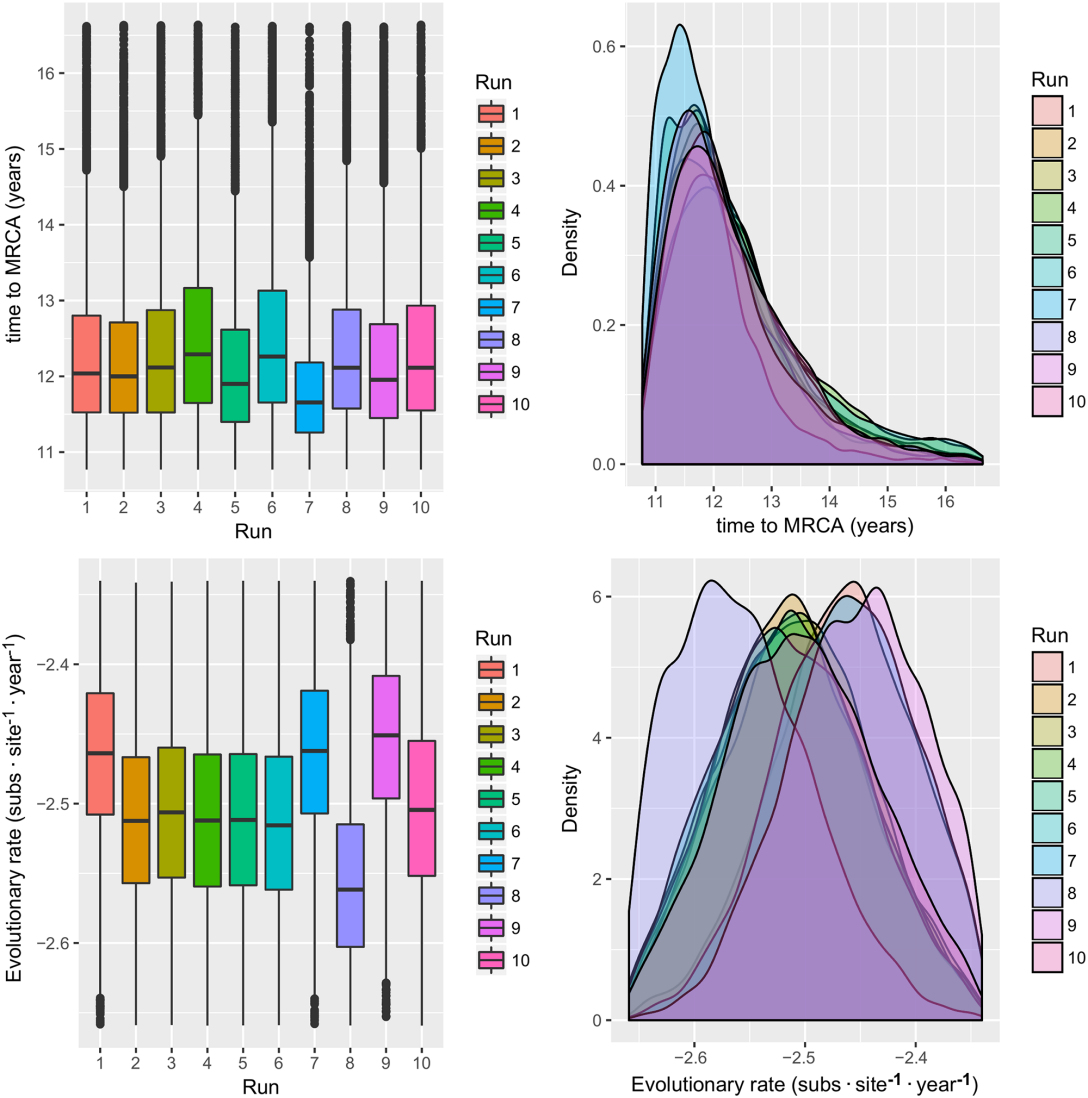
Italian QX genotype MRCA and evolutionary rate. Upper figure: Boxplot (left) and Densityplot of the MRCA posterior probability. Lower figure: Boxplot (left) and Densityplot of the mean evolutionary rate (expressed in base-10 logarithm) posterior probability. Results have been estimated performing ten independent runs based on sequences randomly sampled from the Italian database. The 95HPD intervals are reported for both figures.

**Fig 5:**
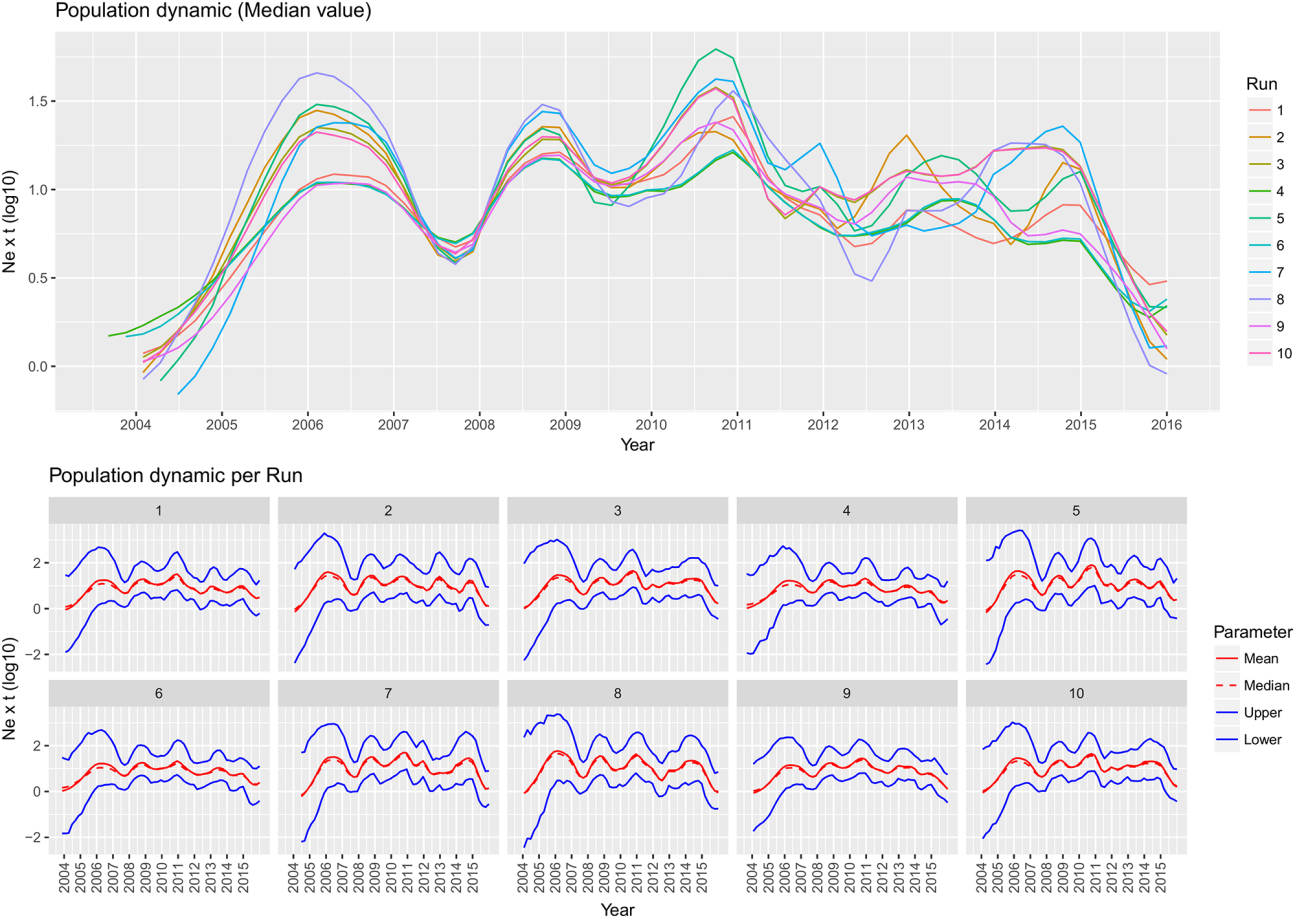
Italian QX genotype population dynamics. Upper figure: Mean relative genetic diversity (Ne x t) of the Italian GI-19 population over time. The results of the ten independent runs have been color-coded. Lower Figure: Mean, median and upper and lower 95HPD values are reported for each run.

Also for this country, BEAST analysis performed on the 10 randomly generated datasets showed concordant results in terms of MRCA, substitution rate (Fig 4) and viral population history (Fig 5). Mean MRCA and substitution rate over the 10 run were 2003,57 [95HPD:2000,82–2005,08] and 3,22·10^−3^ [95HPD:2,33·10^−3^–4,31·10^−3^], respectively. The population dynamics estimation showed a pattern comparable with the international databases ones. After QX genotype introduction, a rapid initial raise in the median genetic relative diversity was replaced by consecutive waves that endured until the final decrease, observed at the end of the considered time period.

**Fig 6:**
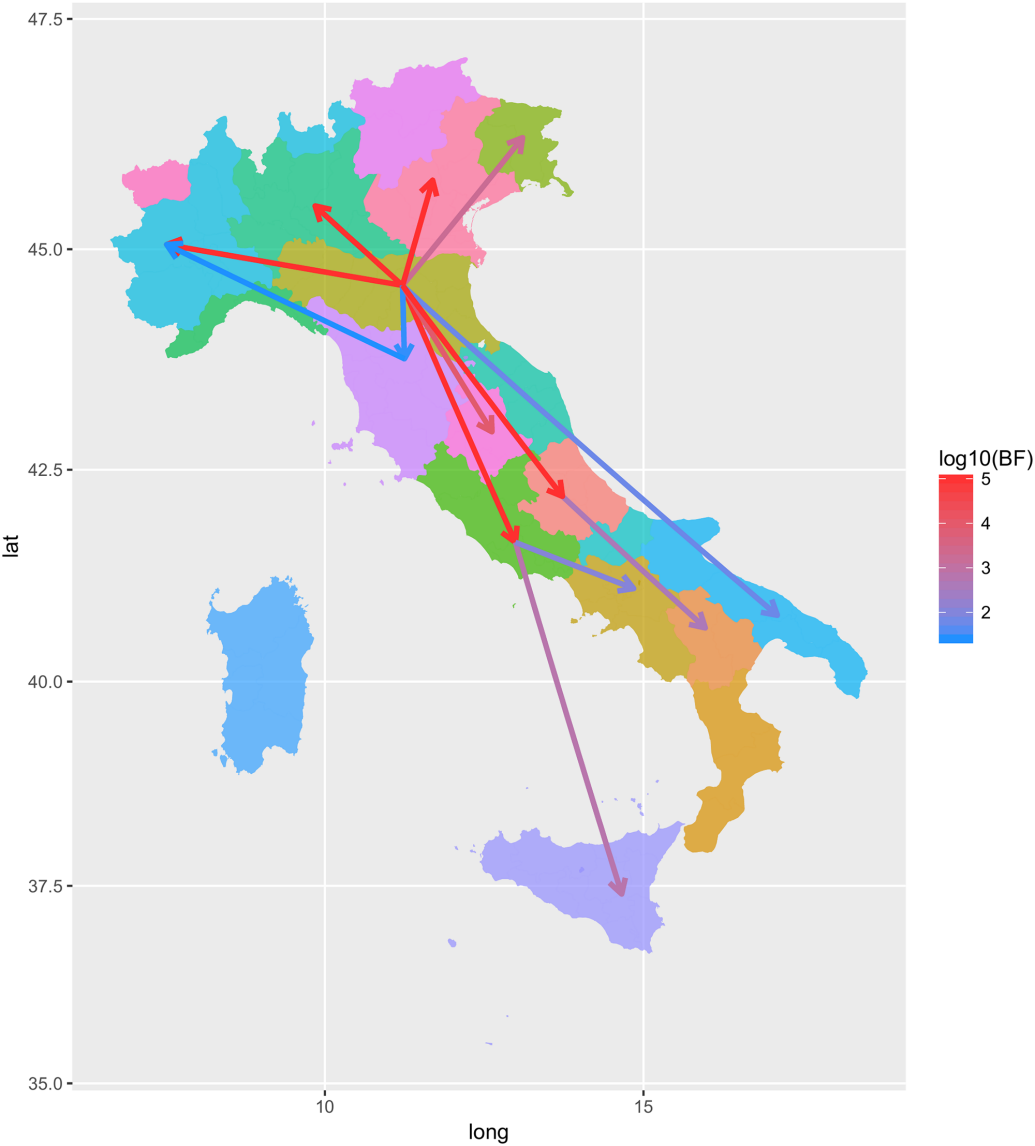
Italian QX genotype migration paths. Well supported migration paths (i.e. BF>20) among Italian regions, which have been colour coded) are depicted. The arrows indicate the directionality of the process while the edge colour is proportional to the base-10 logarithm of the migration rate.

The Italian QX spreading patterns revealed a remarkably complex picture. The Emilia-Romagna was identified as the major source of viral spreading toward both Northern and Southern Italian regions (Fig 6). However, other regions contributed to the viral spreading. More specifically, after the introduction in Emilia-Romagna in 2003 (concordantly with the international dataset estimations), QX radiated to other regions of Northern and Southern Italy following an intricate and hardly predictable pattern. Even if not always significant from a statistical perspective, the migration fluxes among regions were often bidirectional and characterized by multiple introduction events, with regions exporting and importing different strains from different areas. Particularly in the southward diffusion process, the long distance spreading was mediated/facilitated by the passage in intermediate regions (S2 Fig and S2 Video). Similarly to the International scenario, the high densely populated region of Northern Italy showed the highest migration rates (Fig 6).

## Discussion

Since its discovery in 1931 [10], IBV has become one of the major problems for poultry industry around the world and many human and economic resources have been devoted to its control. Nevertheless, the continuous emergence of new variants has frustrated all efforts and compromised the obtained results, in an apparently never ending arm race. The rise and spread of the GI-19 lineage (formerly known as QX genotype) represents an extremely pregnant example in this sense. While the efficacy of available vaccines has been proven against the QX genotype [21,29], the incapability of preventing its diffusion demonstrates that control strategies must not rely only on vaccination. Organizing a coherent and deep knowledge of its evolutionary and epidemiological patterns remains challenging although several epidemiological studies have been performed over time. Additionally, the adequacy of our surveillance and intervention systems should be assessed and improved if necessary, maximizing the rapid detection and control likelihood of new emerging threats. The present study, implementing a phylodynamic approach, tries to consistently investigate these points based on a well-established statistical framework.

It appears clear from this study results that the GI-19 lineage circulated for a long time in China before its recognition as a distinct genotype in 1996 [12]. Concordantly, a sequence published only in 2013 (originating from a sample collected in 1993) demonstrates that this lineage was already present at that time. Our phylodynamic study further backdates the QX emergence of more than ten years. How a usually virulent IBV variant passed undetected for such a long time will require further investigations; however, several non-competing hypothesis can be advocated. At first, only 34 IBV sequences are available from China before 1993 and all originate from samples collected after 1985 (data available at ViPR database [49]), thus a limited surveillance and/or reporting system can have played a major role indeed. At that time, the availability of advanced diagnostic and sequencing technologies was lower if compared with the current ones. Moreover, the backyard poultry farming had and still has a relevant weight in Chinese farming [50]. It is plausible that the first QX strains originated and were allowed to circulate in a rural environment where surveillance systems were less efficient and financial resources more limited [50]. Finally, although the QX genotype emerged as a virulent genotype, there are no proofs that this feature was an ancient one. Other studies dealing with other pathogens have described episodes of virulence increase when mild viruses are allowed to circulated for a prolonged time period in an animal population [51–53].

It can be speculated that low virulence strains circulated undetected or mis/unclassified for several years in the Chinese avian population, potentially becoming more virulent and finally emerging as a major threat for poultry industry. The high evolutionary rate demonstrated in the present study can have favoured this phenomenon, allowing the virus to rapidly explore new phenotypic traits [54,55]. As a side comment, differently from Valastro et al., (2016) [9] who reported the absence of any temporal structure in the whole IBV phylogenesis and the presence of a weak one in the GI-19 one, the present study evidences a higher conformity to the molecular clock theory. When the whole IBV population is considered, it is highly likely that many vaccine derived strains are confused with field ones [26] and included in the analysis, clearly obscuring the temporal structure of the data [39]. Accordingly with this hypothesis, McKinley et al.,[6] had reported that live vaccine use can hamper the accurate measurement of virus mutation rate [6]. Explaining the different results obtained for the GI-19 lineage is far more challenging. Nevertheless, the twofold size of the dataset used in the current study compared to Valastro et al. (2016) [9], and/or the different genomic region considered can account for the different clock-like structure intensity estimated by the two studies.

A parallelism between population size dynamics and the virus geographic spread was evidenced when the QX history was reconstructed. While confined in China, the relative genetic diversity became substantially constant or even decreased after the initial noteworthy expansion (Fig 2). This scenario was bound to change in the late '90, when the lineage was able to invade and spread in other countries. The acquisition of a new niche characterized by a high density and immunological relatively naïve population favoured the emergence of at least four consecutive waves (Fig 2) that substantially corresponded to: QX first introduction in Europe (early 2000), spread in central Europe and Italy (~2005), expansion in Spain and Eastern Europe (~2008–2010) and final invasion of Middle-East and North Africa (2013–2015). However, it must be stressed that the skyline reconstructions are approximations, with an unavoidable degree of uncertainness and relatively broad confidence intervals, such that the median value should be interpreted with cautions [40,43]. Additionally, the viral population size history is representative of the whole GI-19 population at each time period and thus the variations attributable to changes occurring at local scale are probably smoothed or masked by the global scenario. Nevertheless, the coincidence between the areal expansion and the population size changes is at least suggestive and supported by epidemiology theory. Big, high turnover animal concentrations, as the European poultry one is, can create favourable conditions for viral replication and for the genesis of huge viral populations [5]. The dense connections among poultry industries clearly speeded up the GI-19 lineage spreading likelihood among European Union (EU) countries, as testified by the high migration rate that characterizes the within-Europe connections compared with the non-EU ones.

In fact, the analysis of migration history revealed that all European GI-19 strains could have resulted from a single introduction from China (S2 Video and S3 Fig). However, quite surprisingly, even within Europe there is a strong tendency of GI-19 strains to cluster accordingly to the country and, with few exception, the within-country genetic variability can be traced back to one or few introduction events (S1 Video and S3 Fig), suggesting the low propensity of IBV long distance transmission. This evidence conflicts with the sometimes sub-clinical nature of GI-19 infection (particularly in vaccinated chickens) and the prolonged IBV shedding period [56,57].

Despite no unique explanation can be advocated, several factors can have determined the observed scenario. Even if international live poultry trades are quite massive (https://comtrade.un.org/) (S4 Fig), because of EU health legislation, European importations are negligible, reducing the likelihood of strain introduction from developing countries. Additionally, the traded animals are typically young chicks, while field strain infection typically occurs in older animals (about 30 days of age) [21]. Finally, the competition with the earliest established QX strains could have hampered the introduction and settling of new foreign variant of the same genotype. On the other hand, the rapid viral spread within Europe [19,23–25,58] was probably facilitated by the high and unconstrained goods circulation within EU, particularly among geographically neighbouring countries. Nevertheless, the low strain introduction frequency despite the unfavourable epidemiological conditions suggests the feasibility of a more effective fight against IBV, by enhancing the efforts in the direct control measures and through a more integrated cooperation among different areas.

A different picture was depicted when the within country (i.e. Italy) epidemiology was evaluated. The GI-19 lineage was able to rapidly settle in this country after the first introduction, persisting and giving rise to several waves (even if less marked compared with those observed at international scale) until nowadays (Fig 5). Over this timespan, it was able to spread and circulate freely backwards and forwards among closely and distantly related regions (S2 Video and S2 Fig), being the highly densely populated areas of Emilia Romagna the main, but not the only, source of infection (Fig 6). Unfortunately, other studies on IBV spreading routes have not been performed in Italy. The high poultry farm density surely favours the maintenance and circulation of viral infection, particularly in the Northern Italian regions where more than 70% of poultry production is located. However, the transmission of strains among non-neighbouring regions demonstrates that other spreading routes, like animal and supply transportation among different farms of the same poultry production chain, rather than local airborne diffusion are pivotal in the infection dissemination and should thus represent a priority for future actions.

Even if no insight into other countries spreading dynamics was possible, the analysis of the number of lineages that maintain a certain location (i.e. countries) (S1 Video) and the frequent reports present in literature [25], suggest that other countries experimented a similar scenario too.

The first reports of QX genotype (GI-19 lineage) detection in each country typically was closely followed by the introduction time estimated using the phylodynamic approach, proving the good surveillance system efficiency. This evidence is even more relevant considering that the knowledge dissemination about IBV epidemiology is currently based on scientific publications, which imply an unavoidable delay in new outbreak communication. The implementation of more accessible tools for updating and disseminating IBV epidemiological data would be of great benefit for the disease control [59], particularly considering the above mentioned international spreading dynamics.

Despite the efforts made to guarantee the quality of the results, some limitations due to data accessibility must be stressed. At first, the sequence availability is clearly biased toward some Asian, European and Middle East countries, which possess the technological and financial resources to provide advanced diagnostic procedures as well as the willingness to share their outcomes. The severe imbalance in sequence number could bias the results of population parameter estimation and migration pattern reconstruction toward over-represented countries. Additionally, it has been proven by simulation studies that variation in population sizes reconstructed by Bayesian skyline family methods can be subject to spurious phenomena caused by nothing more than the choice of samples [40]. To mitigate both these effects, a down-sampling strategy was applied, balancing the number of strains collected from each area over time. Additionally, by repeating the analyses with different randomly selected sample sets, as suggested by Hall et al.,(2016) [40], the robustness of the obtained results was confirmed.

The lack of information relative to some geographical areas, especially during the initial phase of QX spread, due to either the molecular data paucity or to the availability of sequences obtained from other genomic regions (which precluded their inclusion in the current study) was clearly more problematic and impossible to circumvent with technical expedients. Russia and other Eastern countries represent a paradigmatic example in this sense. Firstly reported in Russia in 2001[13], the QX genotype was proven to persistently circulate in this country [14], which could have played a major role in its dissemination to Europe because of its “intermediate position” between China and the EU in a geographical, commercial and cultural sense.

Even if the role of wild migratory birds in IBV spreading in the world is largely unknown and speculative [4], the circulation of IBV related *Gamma-coronaviruses* in wild species has been reported in several studies [60–62] and many of these hosts, some of those showing high prevalence levels, display migratory habits. The countries where GI-19 has been detected lay along four main flyways: the Black Sea-Mediterranean, the East Africa-West Asia, the Central Asia and the East Asia-Australian routes, even if these paths are clearly a simplification and numerous exceptions from the common patterns have been described [63]. Migration connects many bird populations and virus infected birds can transmit their pathogens to other groups that may subsequently bring it to new areas. Additionally, during migration, birds aggregate in favourable stopover or wintering sites, creating high density populations where the infectious pressure is potentially higher [63].

Based on these considerations, it is likely that at least some of the migration events depicted in Fig 3 and S1 Video could have been mediated by wild birds, as already proposed by other authors to explain unexpected QX introductions [16,18,64] and supported by rising evidences about wild species' ability to carry the virus over long distances [60]. Unfortunately, the scarce molecular epidemiology information on IBV in wild birds, particularly in high poultry producing countries, and its unsystematic nature hampers any definitive conclusion.

Tentatively, the limited number of new strain introductions in each country demonstrated in the current study suggests that their efficacy as active vectors, if any, is probably low. If this is ascribable to the negligible IBV prevalence in wild birds, to other biological factors or to the low contact frequency between these birds and the industrially raised ones will require further investigations.

Despite not weakening the results herein reported, these limitations leave some “black boxes” in our IBV epidemiology knowledge and highlight once more the need for a systematic, coherent and shared diagnostic approach and reporting system that will be fundamental in the understanding and control of the GI-19 lineage and, more importantly, of the future emerging variants.

## Conclusions

The present study provides an overall picture of IBV GI-19 lineage (previously known as QX genotype) history and epidemiology. After a prolonged undetected circulation in China, it was able to spread to the Old World main poultry producing countries, typically by single introduction events followed by establishment and rapid local expansions. Long distance migration events appear sporadic, likely because of epidemiological factor and health policy combination which seems to prevent the frequent introduction and mixing of different strains in the same country. On the other hand, the Italian experience demonstrates the inefficacy of current control strategies implemented at local scale, claiming the need of a further effort and cooperation among private companies and public institutions aimed to the infection control. The incompleteness of available molecular epidemiology data represents an insurmountable limit which left many questions currently unsolved. Particularly, further studies will be necessary to understand the role of Russia in linking Eastern and Western countries and the part played by wild birds in IBV dissemination.

## Supporting information

S1 Fig Network displaying the well supported GI-19 spreading path among different countries estimated using ten independent BEAST runs. The arrows indicates the directionality of the process while the edge colour is representative of the specific run randomly generated sequence dataset.
(PDF)

S2 Fig Time-scaled maximum clade credibility phylogenetic tree (obtained through BEAST analysis) based on Italian GI-19 dataset. Branches have been colour-coded accordingly with their location trait. Additionally, the more likely estimated ancestral location and age have been annotated nearby the corresponding branch and node, respectively.
(PDF)

S3 Fig Time-scaled maximum clade credibility phylogenetic tree (obtained through BEAST analysis) based on international GI-19 dataset. Branches have been colour-coded accordingly with their location trait. Additionally, the more likely estimated ancestral location has been annotated nearby the corresponding node. For representation easiness only results of Run2 are reported.
(PDF)

S4 Fig Map reporting the live-poultry trade network among countries included in the current study. The edge colour is proportional to the base-10 logarithm mean commercial value (reported in USD) of chickens exchanged in the period between 2001–2015. The location of each country has been matched with its centroid.
(PDF)

S1 Video Worldwide spreading dynamics of the IBV GI-19 lineage over time. The MCC phylogeny tree was partitioned in 40 intervals. The circular polygons are proportional to the number of branches over which no trait state transition has occurred (e.g. no change in location between the branch’s parent and child node) in the considered time interval. Branches have been colour coded according with their age from the most ancient (black) to the most recent ones (red). The location of each country has been matched with its centroid.
(GIF)

S2 Video Italian spreading dynamics of the IBV GI-19 lineage over time. The MCC phylogeny tree was partitioned in 40 intervals. The circular polygons are proportional to the number of branches over which no trait state transition has occurred (e.g. no change in location between the branch’s parent and child node) in the considered time interval. Branches have been colour coded according with their age from the most ancient (black) to the most recent ones (red). The location of each Italian region has been matched with its centroid.
(GIF)

S1 Table Table reporting the list of sequences included in the international dataset (accession numbers and related metadata). (XLSX)

S2 Table Table reporting the list of sequences included in the Italian dataset (accession numbers and related metadata). (XLSX)
