# Supplementary figures and images for: Think globally, act locally: Phylodynamic reconstruction of infectious bronchitis virus (IBV) QX genotype (GI-19 lineage) reveals different population dynamics and spreading patterns when evaluated on different epidemiological scales
Source: PLoS One. 2017 Sep 7;12(9):e0184401. doi: 10.1371/journal.pone.0184401 (PMC5589226; doi:10.1371/journal.pone.0184401)

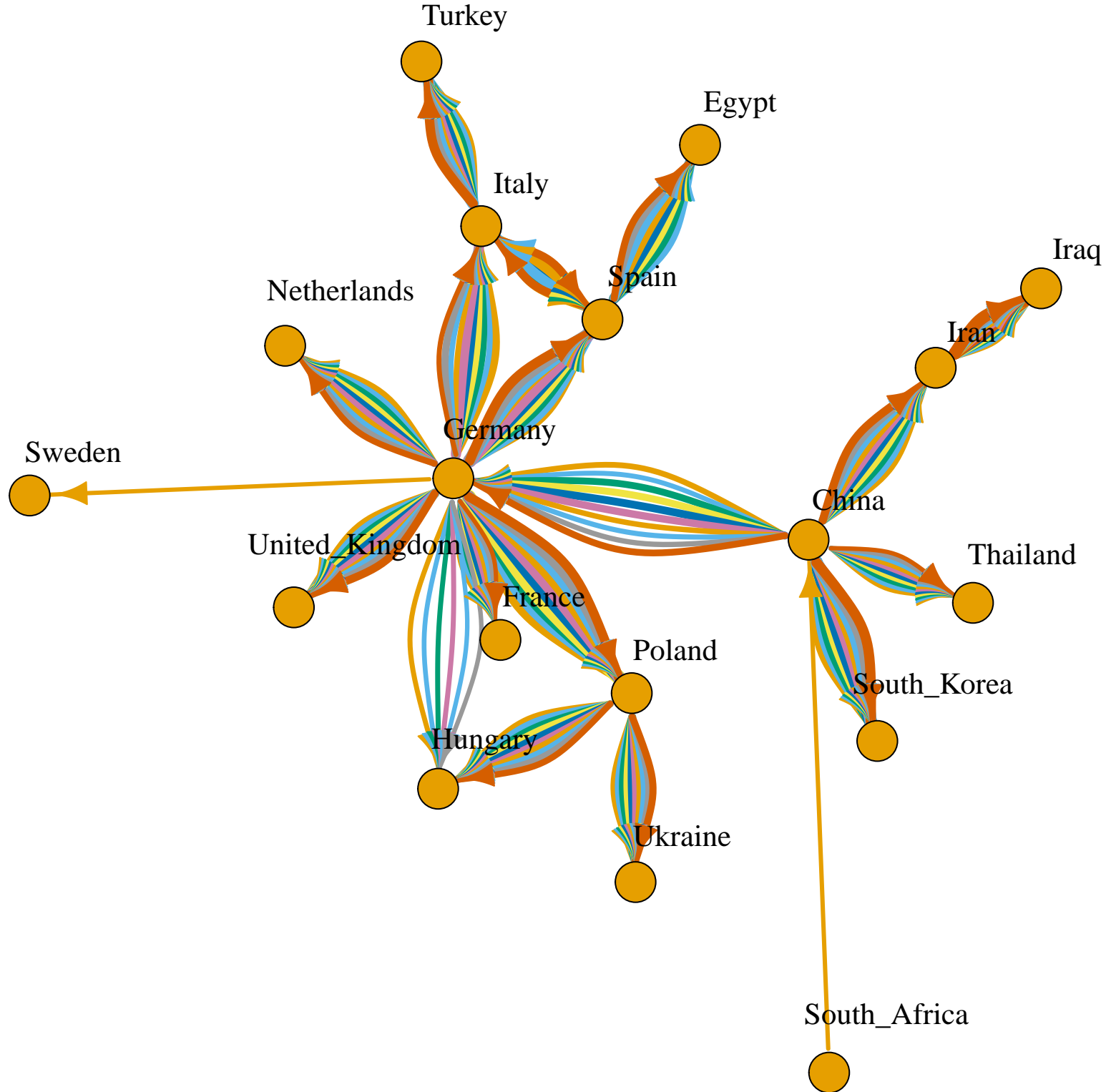

Supplement: S1 Fig — The arrows indicates the directionality of the process while the edge colour is representative of the specific run randomly generated sequence dataset. (PDF) [file pone.0184401.s001.pdf]

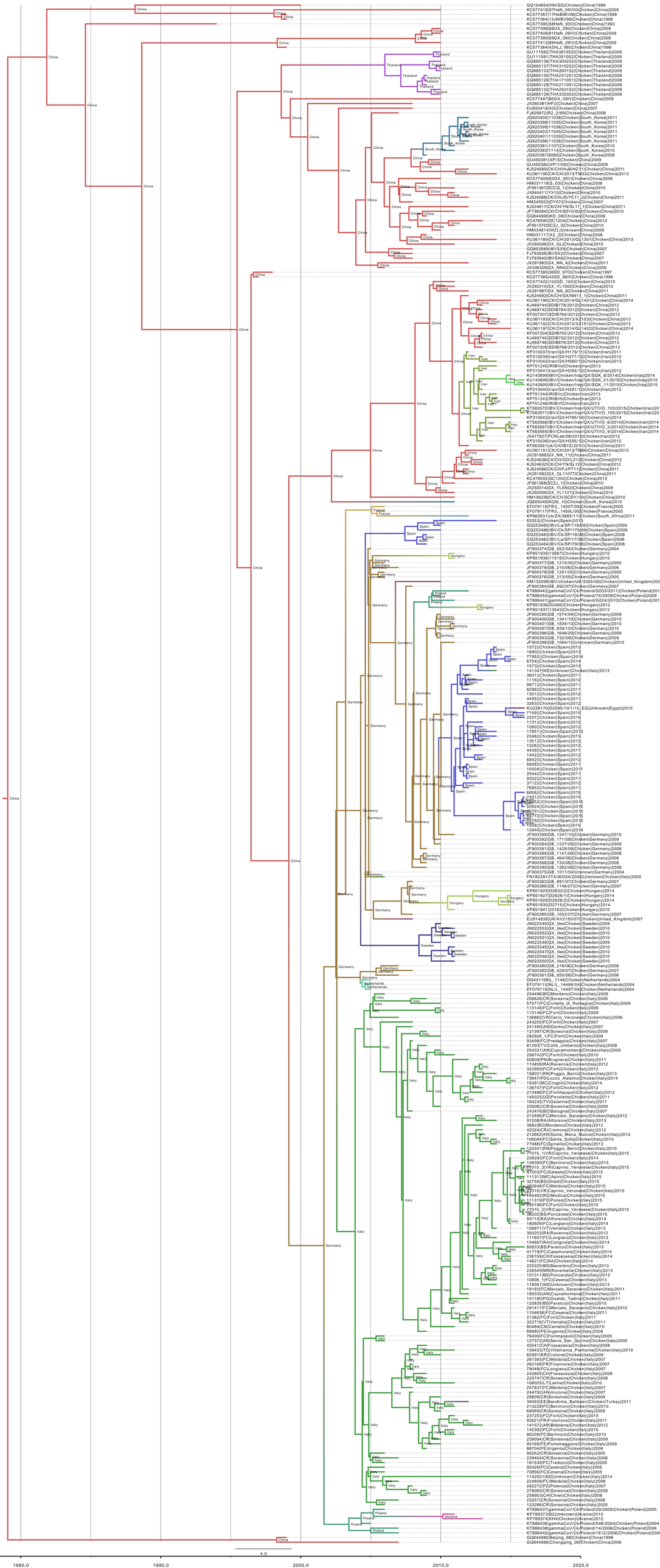

Supplement: S3 Fig — Branches have been colour-coded accordingly with their location trait. Additionally, the more likely estimated ancestral location has been annotated nearby the corresponding node. For representation easiness only results of Run2 are reported. (PDF) [file pone.0184401.s003.pdf]

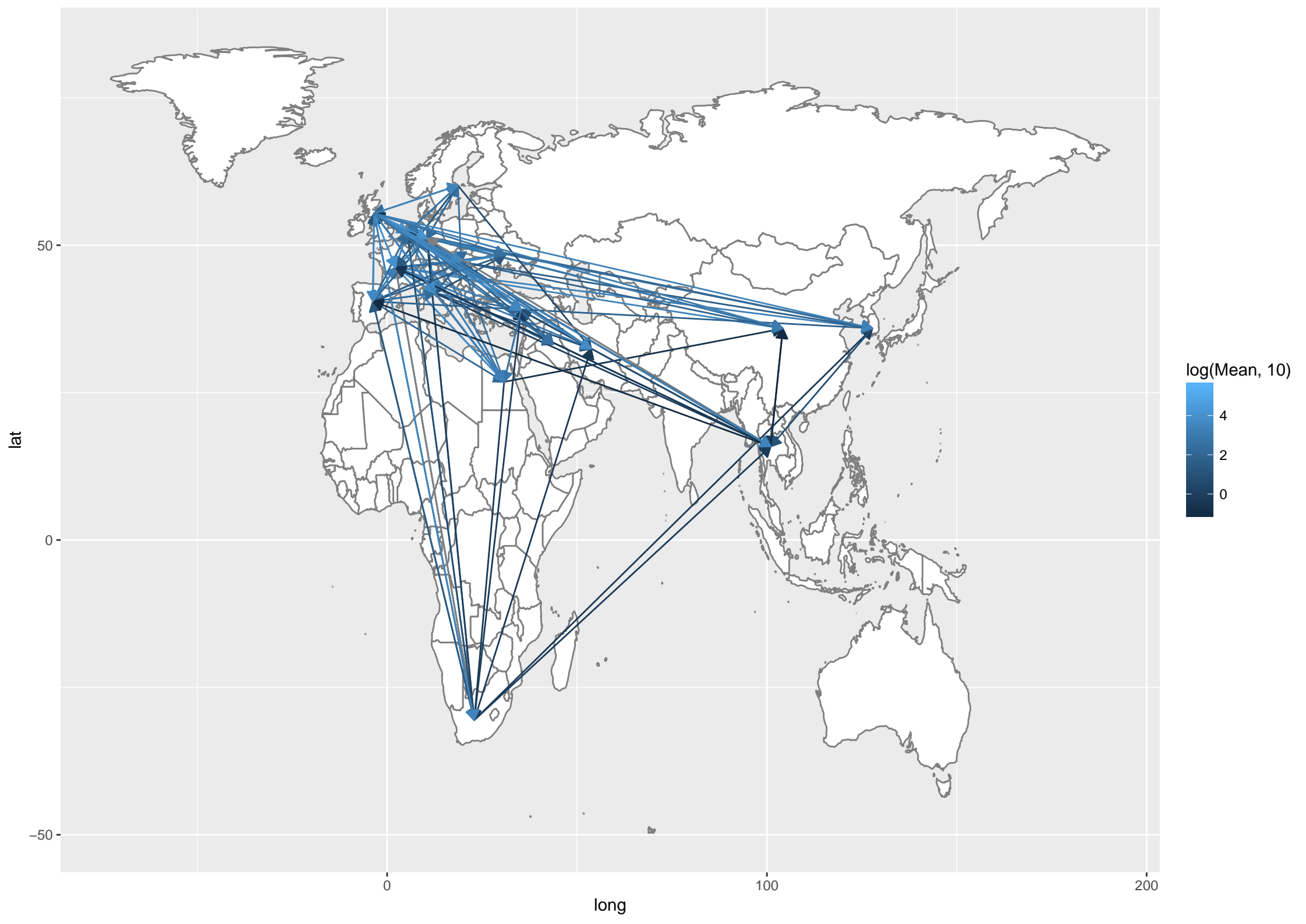

Supplement: S4 Fig — The edge colour is proportional to the base-10 logarithm mean commercial value (reported in USD) of chickens exchanged in the period between 2001–2015. The location of each country has been matched with its centroid. (PDF) [file pone.0184401.s004.pdf]

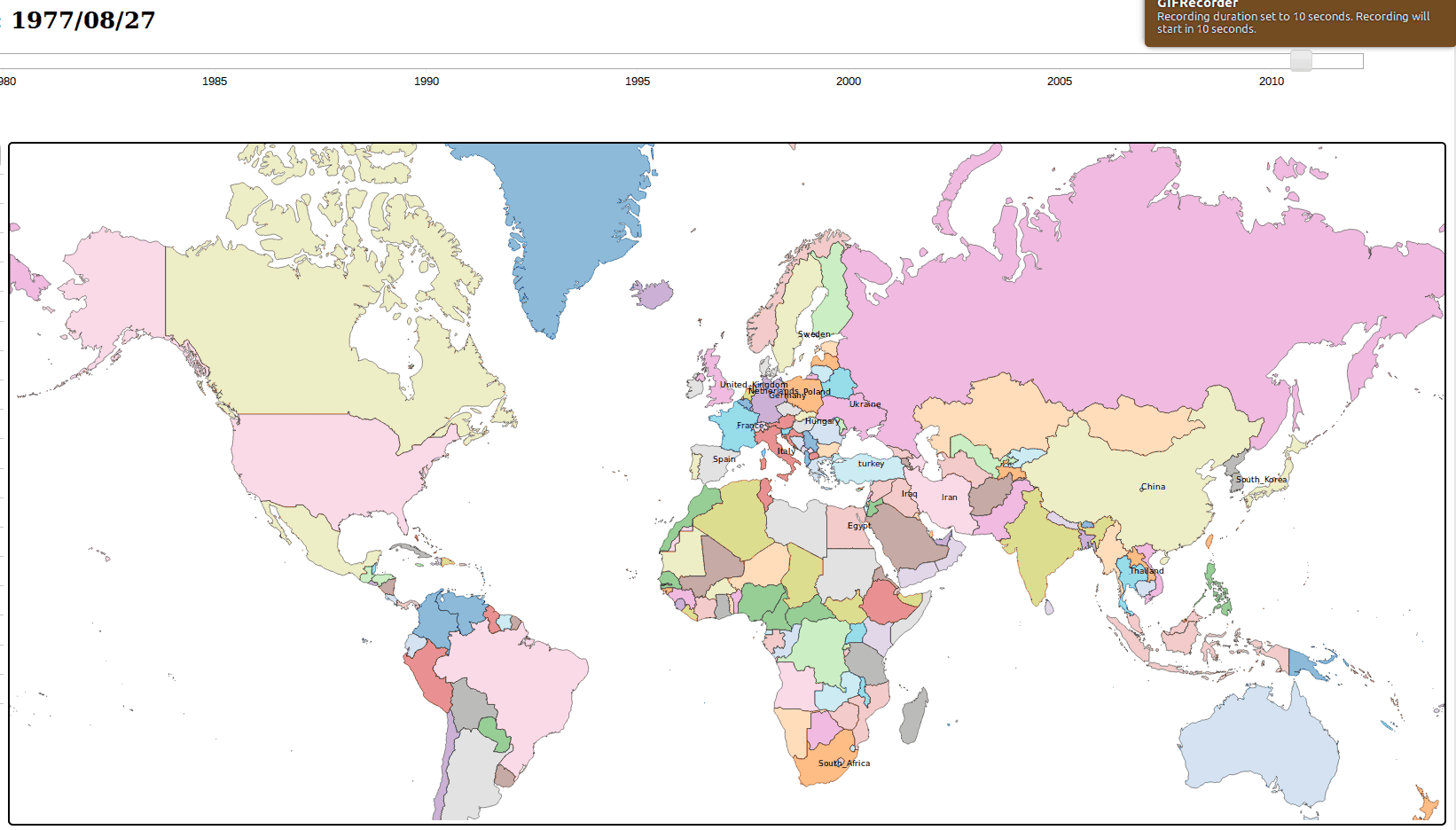

Supplement: S1 Video — The MCC phylogeny tree was partitioned in 40 intervals. The circular polygons are proportional to the number of branches over which no trait state transition has occurred (e.g. no change in location between the branch’s parent and child node) in the considered time interval. Branches have been colour coded according with their age from the most ancient (black) to the most recent ones (red). The location of each country has been matched with its centroid. (GIF) [file pone.0184401.s005.gif]

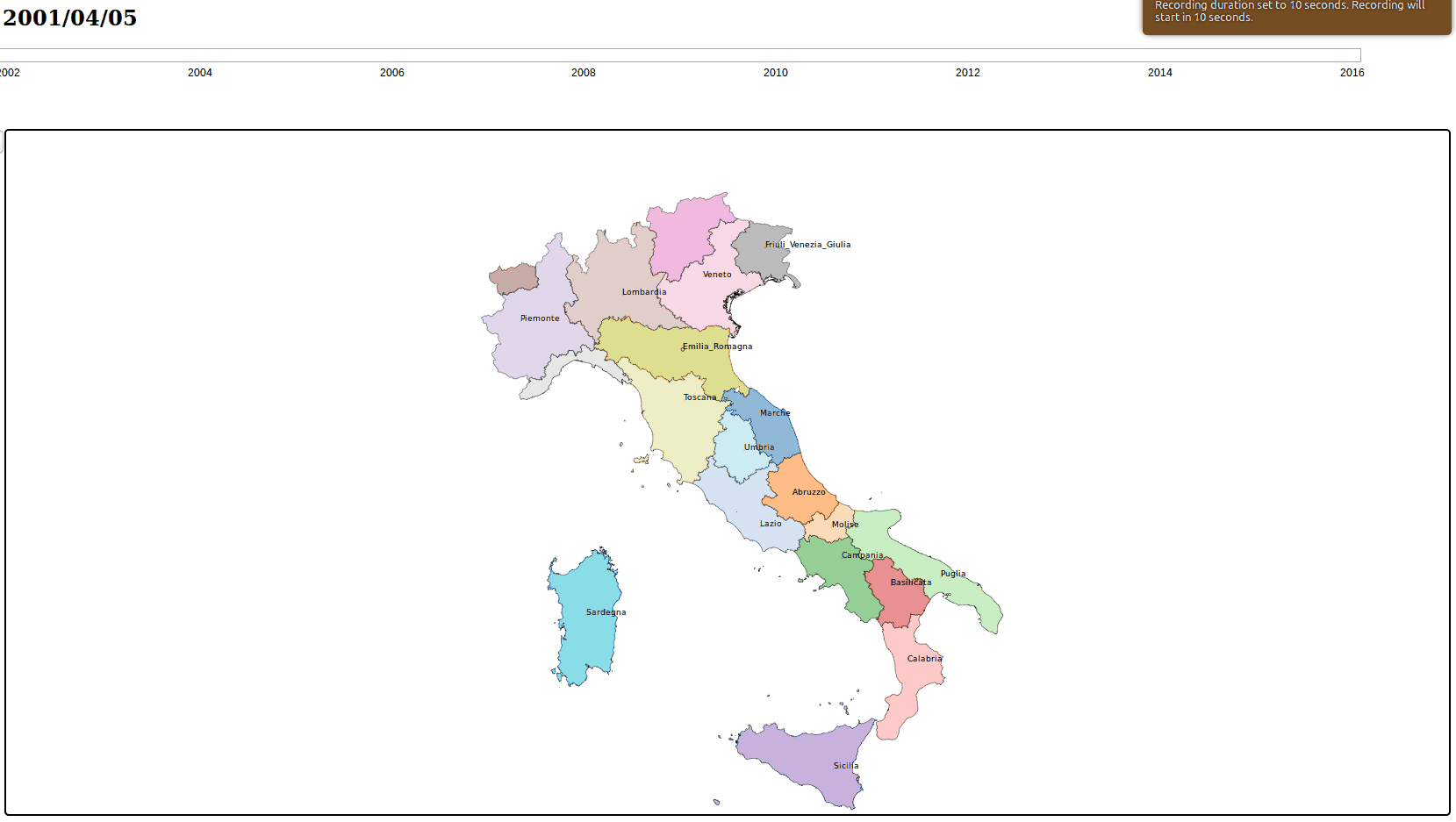

Supplement: S2 Video — The MCC phylogeny tree was partitioned in 40 intervals. The circular polygons are proportional to the number of branches over which no trait state transition has occurred (e.g. no change in location between the branch’s parent and child node) in the considered time interval. Branches have been colour coded according with their age from the most ancient (black) to the most recent ones (red). The location of each Italian region has been matched with its centroid. (GIF) [file pone.0184401.s006.gif]
