# Supplementary material for: Think globally, act locally: Phylodynamic reconstruction of infectious bronchitis virus (IBV) QX genotype (GI-19 lineage) reveals different population dynamics and spreading patterns when evaluated on different epidemiological scales
Source: PLoS One. 2017 Sep 7;12(9):e0184401. doi: 10.1371/journal.pone.0184401 (PMC5589226; doi:10.1371/journal.pone.0184401)

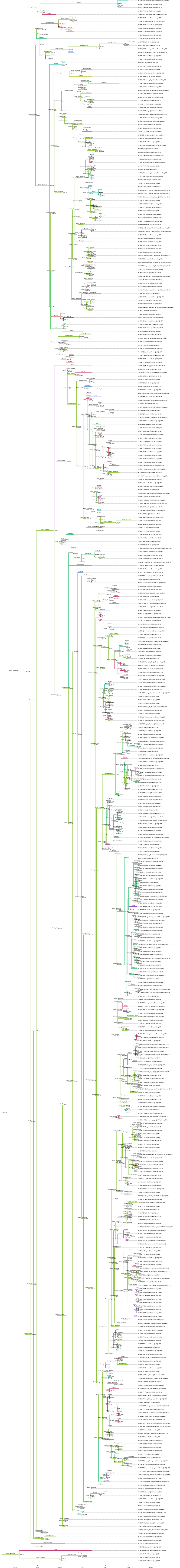

Supplement: S2 Fig — Branches have been colour-coded accordingly with their location trait. Additionally, the more likely estimated ancestral location and age have been annotated nearby the corresponding branch and node, respectively. (PDF) [file pone.0184401.s002.pdf]
